# Supplementary material for: Microparticle-associated tissue factor activity correlates with the inflammatory response in septic disseminated intravascular coagulation patients
Source: PeerJ. 2024 Jan 8;12:e16636. doi: 10.7717/peerj.16636 (PMC10782946; doi:10.7717/peerj.16636)
Supplement: Supplemental Information 2 [file peerj-12-16636-s002.pptx]

## Slide 1
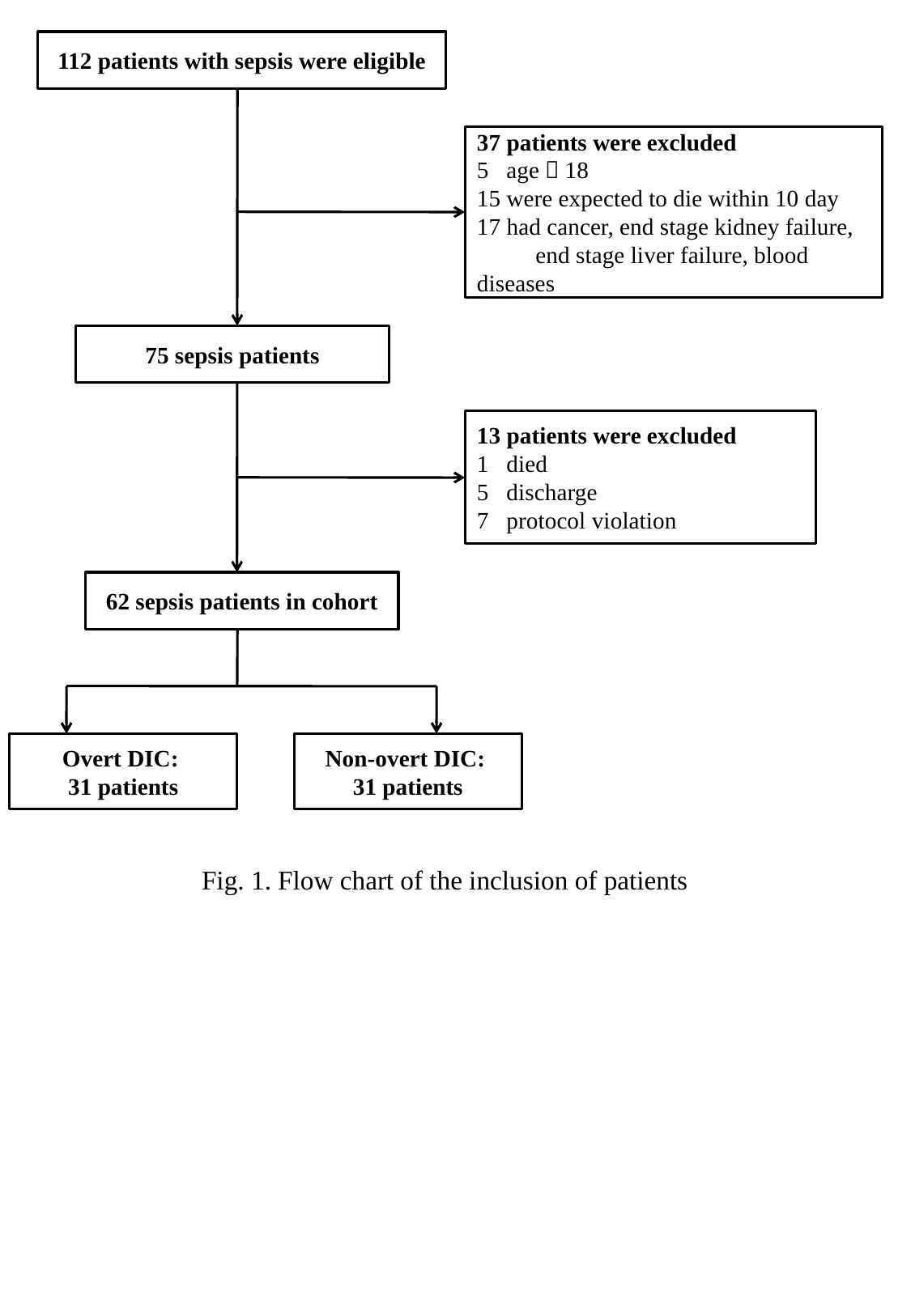

112 patients with sepsis were eligible
37 patients were excluded
5 age＜18
15 were expected to die within 10 day
17 had cancer, end stage kidney failure, end stage liver failure, blood diseases
75 sepsis patients
13 patients were excluded
1 died
5 discharge
7 protocol violation
62 sepsis patients in cohort
Overt DIC:
31 patients
Non-overt DIC:
31 patients
Fig. 1. Flow chart of the inclusion of patients
